# The Effect of Forest Habitats on the Traits and Demographic Structure of *Cardamine bulbifera* (Brassicaceae) Populations

**DOI:** 10.3390/plants14182899

**Published:** 2025-09-18

**Authors:** Laurynas Taura, Zigmantas Gudžinskas

**Affiliations:** Laboratory of Flora and Geobotany, State Scientific Research Institute Nature Research Centre, Žaliųjų Ežerų Str. 47, LT-08412 Vilnius, Lithuania

**Keywords:** conservation, forest habitats, immature individuals, juvenile individuals, mature individuals, population density, vegetative reproduction

## Abstract

The conservation of plant species requires detailed knowledge of their reproductive behaviour and population demographic structure. This is particularly important for species such as *Cardamine bulbifera*, which depend on old-growth forest habitats and rely predominantly or entirely on vegetative reproduction through axillary bulbils. Although *C. bulbifera* has a wide native range, little is known about its population structure and dynamics. The aim of this study was to assess the demographic composition, density and main traits of *C. bulbifera* individuals in six populations occurring in three types of forest habitats in southern Lithuania: Fennoscandian hemiboreal natural old broadleaved deciduous forests, Fennoscandian herb-rich forests with *Picea abies* and *Galio-Carpinetum* oak–hornbeam forests. Field studies were conducted in 2023, during which a total of 20 sampling plots (each 1 m^2^) were analysed in each population, arranged in a transect. The study revealed an absolute dominance of young (juvenile and immature) individuals in the populations (89.2%), whereas mature individuals comprised only a small fraction (10.8%). The proportion of mature individuals was significantly larger in hornbeam forests than in the other two forest types. The highest density of individuals was recorded in broadleaved forest, while the lowest density was found in spruce forest habitat. Mature *C. bulbifera* individuals in hornbeam habitats were significantly taller and had longer inflorescences than those in other habitats. The highest mean number of bulbils was produced by individuals of the studied species in spruce habitats, while bulbil production was lowest in hornbeam habitats. The strongest negative contribution to the number of *C. bulbifera* individuals was the area of bare soil in the sampling plot, whereas herb cover had the strongest positive effect. These results highlight habitat-specific differences in *C. bulbifera* population structure and suggest that the long-term viability of its populations is closely associated with forest type, as well as stability of the habitat and plant community. The optimum habitat conditions for *C. bulbifera* are found in old broadleaved forests, and habitats with natural succession are the most favourable for its growth and conservation.

## 1. Introduction

The decline in the frequency or number of populations of many plant species within their native range is accelerating at an increasing rate across all continents and almost all biomes [1,2]. Nevertheless, this phenomenon, which is induced by habitat destruction and climate change resulting from human activities [3,4], is well documented and the consequences of this decline are well understood and widely discussed within the scientific community [5,6]. Aiming to halt the massive decline in many plant and other species, a wealth of effort and resources are devoted to implementing conservation measures [7,8]. For these efforts to be effective, all conservation measures must be based on comprehensive scientific knowledge- and evidence-based approaches [9].

Many researchers and conservationists are concerned that empirical field research has decreased in extent in recent decades [10,11]. Ensuring species survival requires extensive fieldwork and a thorough understanding of species ecology, demography, regional dynamics, genetics and computational analysis [12,13]. It is essential to understand the ecological interactions of species with other organisms because the loss of these interactions can result in rapid species extinction [9]. The relationship between species traits and ecological interactions highlights the critical role of a species in maintaining ecosystem stability [14,15]. Individual species are essential for maintaining ecosystem stability due to their unique responses to environmental fluctuations, which trigger stabilising mechanisms [16].

Some plant species are at an increased risk of extinction due to their small geographical range, small and fragmented populations, low reproductive potential, or their dependence on specialised habitats, pollinators, or seed dispersal agents [17,18]. Each species is unique and plays a vital role in global biodiversity [17,19]. The loss of a single species, particularly a plant species, can trigger cascading effects within ecosystems, which can potentially impact their functioning and stability [9,17,18,19].

Forest habitats around the world are being intensively exploited for timber [20,21]. While the deforestation of tropical forests is well documented and has become a global problem, forests in temperate regions are also being replaced by species-poor tree plantations, which leads to a loss of biodiversity [22,23]. The species of plants and other organisms that are adapted to natural habitats which have existed for centuries and changed slowly are the most affected when old or mature forest stands are cleared. Old-growth forests contain a particular species diversity due to specific conditions, an equilibrium between stability and natural disturbances, and a variety of microhabitats [24,25]. The fungi, mosses and insects that are characteristic of old-growth forests are particularly affected [26,27], as are vascular plants that reproduce slowly or whose biological characteristics severely restrict their spread into new areas [28]. Dozens of plant species inhabiting European boreal and temperate forests (e.g., *Cardamine bulbifera* (L.) Crantz, *Cypripedium calceolus* L., *Epipogium aphyllum* Sw., *Hemipilia cucullata* (L.) Y. Tang, H. Peng & T. Yukawa, *Hordelymus europaeus* (L.) Jess. ex Harz, *Melittis melissophyllum* L., *Pulsatilla patens* (L.) Mill.) are considered threatened or endangered in at least some regions [29,30,31,32,33].

*Cardamine bulbifera* is a typical vernal woodland plant that reaches its peak of development and flowering before the tree crowns form a dense canopy. It is strongly associated with valuable ancient woodland habitats and is considered an indicator species of these habitats [34,35]. Throughout its range, *C. bulbifera* grows mainly in deciduous broadleaved or mixed coniferous forests. In most of Western and Central Europe, *C. bulbifera* is closely associated with forests dominated by *Fagus sylvatica*. In Southern Europe, it is found in mountain forests, typically on north-facing slopes [36,37,38]. It thrives in both wet and mesic habitats with acidic or alkaline soils. Occasionally, this species can be found on shady stream sides and in forest glades [37]. In Lithuania, it primarily grows in mixed broadleaved and hornbeam forests, as well as in mixed spruce forests [31,39].

Studies of the development and cytogenetics of *C. bulbifera* have revealed that only a small proportion of flowers produce fruit, with fewer than two percent of zygotes progressing beyond the early stages of embryogenesis [34]. This dodecaploid species (2n = 12x = 96) has therefore almost completely lost the ability to reproduce sexually, relying solely on vegetative reproduction through bulbils that develop in leaf axils [35]. Phylogenomic studies suggest that *C. bulbifera* arose from the diploid Caucasian endemic *C. abchasica* Govaerts around 20,000 years ago during the Last Glacial Maximum [35]. Its post-glacial range is thought to have formed almost entirely through clonal reproduction, with water and animals likely being the main vectors of bulbil dispersal [37,38].

The main threats to *C. bulbifera* in Europe are the clearance of forests, the eutrophication of habitats, climate change and contemporary changes in forestry practices, such as replacing natural forests with intensive plantations of native coniferous trees or various introduced species [31,37,38]. Despite the existence of large *C. bulbifera* populations in Lithuania, this species has decreased significantly or become extinct in several localities in recent decades [31,39].

Despite the wide distribution of *C. bulbifera*, little is known about the recruitment rate and demographic structure of its populations. Information on the viability and germination of bulbils is scarce and somewhat controversial. Some authors claim that bulbils germinate poorly and recruitment is slow [40], whereas others indicate intense reproduction and fast germination after bulbils reach the ground [37]. It has been reported that, once they have taken root, fallen bulbils produce the first leaf in the second year, after which young individuals develop a characteristic basal leaf. In the third or fourth year of growth, it develops an aerial shoot [37]. We were unable to locate published data on the demographic structure of *C. bulbifera* populations.

This study aimed to determine the state of *C. bulbifera* populations in three main habitat types, assessing the demographic structure and traits of individuals. The following questions were addressed in this study: 1. What is the composition of populations according to maturity groups, and how does this depend on habitat type? 2. What is the density of individuals in different maturity groups, and how does this depend on habitat type? 3. Do traits and reproductive potential vary depending on habitat type? 4. How do community structure, species composition, and diversity influence the abundance and density of *C. bulbifera* individuals of different maturity groups? 5. What habitat conditions ensure the long-term survival of *C. bulbifera* populations?

## 2. Materials and Methods

### 2.1. Study Species

*Cardamine bulbifera* (L.) Crantz (*Dentaria bulbifera* L.; Brassicaceae) is a perennial plant with a thick rhizome. The stem is upright and unbranched, reaching a height of 40–70 cm. Young (juvenile and immature) plants produce a trifoliate or pinnatifid leaf. The lower stem leaves of flowering plants are pinnatifid and gradually reduced to simple lanceolate leaves towards the apex of the stem. The plant forms bulbils in the axil of the stem leaves and in the inflorescence. It flowers in May in Lithuania. The flowers are light or dark pink to almost white. The siliquas are narrow and rarely fully develop, and even more rarely produce seeds [34,35,37]. *Cardamine bulbifera* reproduces by bulbils. It grows in deciduous and mixed forests, usually in fertile, moderately moist, calcareous soils [37].

*Cardamine bulbifera* has a wide range, extending from Western to Central and Eastern Europe, northwards to southern Scandinavia and eastwards to Western Siberia and Southwest Asia. In the Netherlands and Ireland, it is considered a naturalised alien species [34,37,38]. In Lithuania, *C. bulbifera* is a rare, protected species. Most of its localities are concentrated in the southern and central parts of the country, with isolated localities in the west and east [39]. The Baltic countries are at the northeastern limit of the *C. bulbifera* range [41].

### 2.2. Study Sites

**Figure 1:**
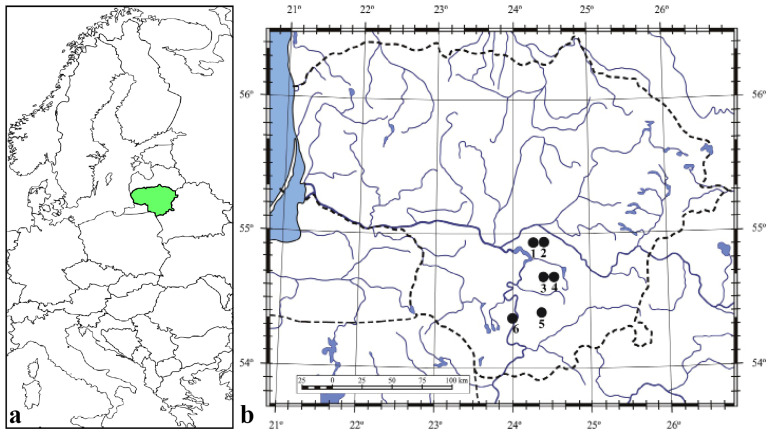
Location of Lithuania (green) in Europe (**a**) and study sites of *Cardamine bulbifera* populations (**b**): 1. Šešuva; 2. Pašuliai; 3. Sutartiškės; 4. Kaukinė; 5. Kalesninkai; 6. Vidzgiris.

Six *C. bulbifera* populations in southern Lithuania were selected for this study (Figure 1; Table 1). These populations were selected according to the following criteria: they must occupy an area of at least 1000 m^2^ (0.1 ha) within a homogeneous habitat, with individuals dispersed relatively evenly over the habitat. Habitat types were identified following the Interpretation Manual of European Union Habitats (version EUR 28) [42]. The study sites were selected from three EU habitat types: Fennoscandian hemiboreal natural old broadleaved deciduous forests (hereafter referred to as ‘broadleaved forest’), Fennoscandian herb-rich forests with *Picea abies* (hereafter referred to as ‘spruce forest’) and *Galio-Carpinetum* oak–hornbeam forests (hereafter referred to as ‘hornbeam forest’). Two populations were selected for study in each habitat type (Table 1). The studied populations were named after the nearest village or other geographical landmark, such as a river or forest.

Šešuva: This site was selected in the Šešuva Botanical Reserve in Pravieniškės Forest (Kaišiadorys district), in a broadleaved forest habitat. The dominant tree species were *Quercus robur* L., *Fraxinus excelsior* L. and *Acer platanoides* L. Some *Quercus robur* individuals were over 250 years old. The shrub layer was quite dense (Table 2), dominated by *Corylus avellana* L. The relatively dense herb layer consisted of species characteristic of broadleaved and mixed forests, including *Aegopodium podagraria* L., *Anemone nemorosa* L., *Asarum europaeum* L., *Galium odoratum* (L.) Scop., *Hepatica nobilis* Mill., *Lamium galeobdolon* (L.) L., *Lathyrus vernus* (L.) Bernh., *Mercurialis perennis* L., *Paris quadrifolia* L., *Ranunculus cassubicus* L., *Sanicula europaea* L. and *Stellaria holostea* L. The bryophyte layer was sparse and covered about 10% of the surface (Table 2).

Pašuliai: This site was selected in the Šešuva Botanical Reserve in Pravieniškės Forest (Kaišiadorys district), in a spruce forest habitat. The dominant species in the tree layer were *Picea abies* (L.) H. Karst. and *Betula pendula* Roth. The shrub layer was sparse, consisting mainly of solitary *Corylus avellana* shrubs and *Picea abies* young trees. The herb layer was relatively sparse and consisted of species characteristic of spruce forests: *Anemone nemorosa*, *Asarum europaeum*, *Carex digitata* L., *Hepatica nobilis*, *Lamium galeobdolon*, *Luzula pilosa* (L.) Willd., *Maianthemum bifolium* (L.) F.W. Schmidt, *Moehringia trinervia* (L.) Clairv., *Oxalis acetosella* L., *Pulmonaria obscura* Dumort. and *Scrophularia nodosa* L. The sparse bryophyte layer (Table 2) consisted mainly of small patches of *Eurhynchium angustirete* (Broth.) T.J. Kop., *Atrichum undulatum* (Hedw.) P. Beauv. and *Rhodobryum roseum* (Hedw.) Limpr.

Sutartiškės: This site was selected in the Kaukinė Botanical Reserve in the Kaukinė Forest (Kaišiadorys district), in a broadleaved forest habitat. The dominant tree species were *Tilia cordata* Mill. and *Quercus robur*, while the subdominant species were *Acer platanoides* and *Betula pendula*. The sparse shrub layer (Table 2) was mainly composed of *Corylus avellana* shrubs and young *Tilia cordata* and *Acer platanoides* trees. The herb layer was dense and consisted of species characteristic of broadleaved forests, including *Aegopodium podagraria*, *Anemone nemorosa*, *Corydalis cava* (L.) Schweigg. & Körte, *Ficaria verna* Huds., *Gagea lutea* (L.) Ker Gawl., *Galium odoratum*, *Lamium galeobdolon*, *Paris quadrifolia*, *Pulmonaria obscura* and *Ranunculus lanuginosus* L. and *Stellaria holostea*. The sparse bryophyte layer consisted of unevenly dispersed patches of *Atrichum undulatum*, *Eurhynchium angustirete* and *Plagiomnium undulatum* (Hedw.) T.J. Kop.

Kaukinė: This site was selected in the Kaukinė Botanical Reserve in the Kaukinė Forest (Kaišiadorys district) in a spruce forest habitat. The dominant species in the tree layer were *Picea abies* and *Betula pubescens* Ehrh. (Table 2). The moderately dense shrub layer was mainly composed of *Corylus avellana* shrubs and young *Picea abies* trees. The herb layer was abundant and consisted of species characteristic of mixed spruce forests, including *Anemone nemorosa*, *Asarum europaeum*, *Galium odoratum*, *Hepatica nobilis*, *Lamium galeobdolon*, *Maianthemum bifolium*, *Milium effusum* L., *Oxalis acetosella*, *Stellaria holostea*, *Stellaria nemorum* L. and *Viola reichenbachiana* Jord. ex Boreau. The bryophyte layer was sparse and consisted mainly of small patches of *Eurhynchium angustirete* and *Plagiomnium undulatum.*

Kalesninkai: This site was selected in the Kalesninkai Forest (Alytus district) in a hornbeam forest habitat. The dominant species in the tree layer was *Carpinus betulus* L., with *Picea abies* as the subdominant species (Table 2). The sparse shrub layer (covering 10%) consisted of solitary individuals of *Corylus avellana*, *Carpinus betulus* and *Picea abies*. The herb layer was also relatively sparse, composed mainly of species characteristic of hornbeam forests, including *Aegopodium podagraria*, *Asarum europaeum*, *Carex sylvatica*, *Cardamine flexuosa* With., *Ficaria verna*, *Galium odoratum*, *Lamium galeobdolon*, *Pulmonaria obscura*, *Sanicula europaea* and *Stellaria holostea*. The bryophyte layer was sparse, consisting of small patches of *Eurhynchium oedipodium* (Mitt.) Jaeg. and *Plagiomnium undulatum*. Approximately 40% of the habitat surface was bare soil.

Vidzgiris: This site was selected in the Vidzgiris Botanical Reserve, in the Vidzgiris Forest (Alytus district), in a hornbeam forest habitat. The dominant species in the tree layer were *Carpinus betulus*, *Quercus robur* and *Tilia cordata* (Table 2). The shrub layer was moderate and mainly composed of young *Carpinus betulus* and *Corylus avellana* individuals. The relatively sparse herb layer consisted of species characteristic of hornbeam forests, including *Aegopodium podagraria*, *Anemone nemorosa*, *Asarum europaeum*, *Ficaria verna*, *Hepatica nobilis*, *Lamium galeobdolon*, *Paris quadrifolia*, *Phyteuma spicatum* L., *Polygonatum multiflorum* (L.) All., *Pulmonaria obscura* and *Stellaria holostea*. The sparse bryophyte layer consisted only of small patches of *Plagiomnium undulatum* and *Atrichum undulatum*. About half of the habitat surface was bare soil.

Analysis of soil samples collected from each study site revealed that the soil at the Kaukinė and Sutartiškės sites was extremely acidic, while at Pašuliai it was very strongly acidic. The soil at the Kalesninkai site was strongly acidic, and at the Šešuva and Vidzgiris sites it was slightly acidic (Appendix A, Table A1). The phosphorus content of the soil varied greatly between sites, ranging from low at Sutartiškės to moderate at Šešuva and Vidzgiris and high at Pašuliai, Kaukinė and Kalesninkai. Content of potassium in the soil was low at most sites, except at the Kaukinė site, where it was moderate, and at the Kalesninkai site, where it was very high. Total soil nitrogen content was low at the Pašuliai, Sutartiškės and Kaukinė sites, whereas at the Šešuva, Kalesninkai and Vidzgiris sites it was moderate. Soil humus content was low at the Pašuliai site, moderate at the Sutartiškės, Kaukinė and Kalesninkai sites, and high at the Šešuva and Vidzgiris sites (Appendix A, Table A1).

### 2.3. Field Survey Procedures

Field surveys were conducted at the six selected study sites in the first decade of May 2023, during the mass flowering of *Cardamine bulbifera*. In the first stage of the assessment of the *C. bulbifera* population, a phytosociological relevé of the plant community in the study area (400 m^2^) was performed, following the Braun–Blanquet [43] approach. Plant diversity and abundance were analysed across each vegetation layer (the first and second tree layers, the shrub layer, the herb layer and the bryophyte layer). Additionally, the coverage of plant debris and bare soil was assessed.

The structure of the *C. bulbifera* population was studied using the sampling plot method. At each study site, 20 sampling plots measuring 1 m^2^ each were set up along a transect running from south to north. To minimise edge effects and ensure habitat uniformity, the transect was initiated at least 3 m from the outer boundary of the *C. bulbifera* stand. The sampling plots were delimited using a wooden frame measuring 1 m on each side, each of which had a measuring scale. The sampling plots within the transect were placed with minimal spacing (5 cm), corresponding to the width of the wooden frame.

During the survey of each sampling plot, the tallest plant was measured using a tape measurer. The mean height of the herb layer was determined by taking measurements of the herbs at two opposite corners of the sampling plot. All plant species present in the sampling plot (including seedlings and saplings of woody plants, herbaceous plants and bryophytes) were identified and recorded, and their percentage cover was estimated. Species with low coverage (less than 5%) were assessed with a precision of 0.1% (equivalent to 1 cm^2^), whereas coverage of more abundant species was assessed with a precision of 1% (equivalent to 10 cm^2^). Additionally, the cover of plant debris (e.g., dead leaves, branches and bark) and the percentage of bare soil (areas not covered by vegetation or debris) were assessed.

*Cardamine bulbifera* individuals in the sampling plot were thoroughly counted, and their height from the soil surface to the highest point (top of the stem or highest part of the leaf) was measured to a precision of 1 cm. The length of the stem section bearing bulbils of both non-flowering and flowering mature individuals was measured from the lowest leaf containing a bulbil in its axil to the topmost bulbil. The number of bulbils was carefully counted avoiding their detachment from the plant. The length of the inflorescence of flowering mature individuals was measured from the lowest to the highest flower, and the number of flowers was counted.

### 2.4. Maturity Groups of Individuals

**Figure 2:**
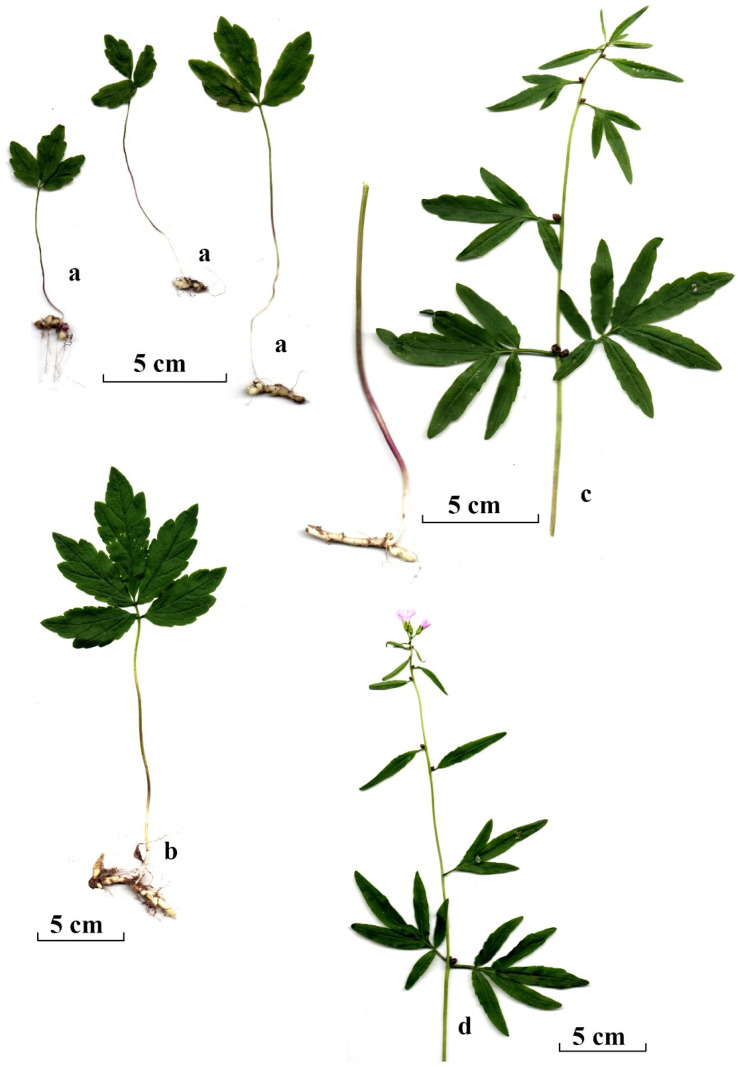
The morphology of *Cardamine bulbifera* individuals in recognised maturity groups and subgroups: (**a**) juvenile individuals; (**b**) immature individual; (**c**,**d**) mature individuals (**c**, non-flowering mature; **d**, flowering mature). Scale bar: 5 cm.

Each *C. bulbifera* plant recorded in the sampling plots was assigned to one of three developmental groups, categorised by plant size and morphological characteristics. These groups were juvenile, immature and mature individuals. Because of significant differences in plant development, the group of mature individuals was further divided into two subgroups: non-flowering and flowering plants. The juvenile group comprised small plants, typically 3–10 cm tall, bearing a single trifoliate leaf. These plants had grown from bulbils in the year of study or the previous year (Figure 2). Plants with a well-developed pinnatifid leaf (occasionally two leaves) but no stem were assigned to the immature group. Plants with a developed stem and bulbils in the axils of the stem leaves were assigned to the mature group. Mature individuals without any fully developed flowers (although sometimes with a few rudimentary flowers) were assigned to the group of non-flowering mature individuals. Plants with a well-developed stem, bulbils in the axils of the stem leaves and at least one fully developed flower were assigned to the group of flowering individuals (Figure 2).

### 2.5. Statistical Analyses

All data sets were subjected to a Shapiro–Wilk test to assess their distribution. Since many of the data sets exhibited non-normal distribution or the samples were of unequal size, non-parametric statistical analysis methods were applied. The Kruskal–Wallis (H value) test was used for comparing multiple data sets, while the Mann–Whitney (U value) post hoc test was used for comparing pairs of data sets. However, due to the presence of zero values in the data sets, Dunn’s post hoc test (*z* value) was used to compare the density of individuals in different maturity groups. Statistically significant differences were defined as those with *p* < 0.05. When presenting the results of a comparative statistical analysis, the actual *p*-values were indicated when *p* > 0.001. Descriptive statistics typically include mean values and standard deviation (mean ± SD). However, when analysing plant traits, the minimum and maximum values are also provided. Descriptive statistical analyses and population comparisons were performed using PAST 5.1 software [44].

The similarity of species composition between the study populations was assessed using the Jaccard index (J). A list of species recorded in all sampling plots of each population was used for comparison, applying a binary (1 or 0) matrix. As the data sets including data from all populations (pooled data sets) were normally distributed, the relationships between them were assessed using linear correlation analysis (Pearson’s *r*).

Principal component analysis (PCA) was used to assess the effect of habitat and community characteristics on the total and individual maturity group density of *C. bulbifera*. Six continuous predictors were used: mean height (cm) of herbs; cover (%) of plant debris; cover (%) of bare soil; total cover (%) of the herb layer; cover (%) of bryophyte layer; and the number of plant species. All variables were centred and scaled to unit variance, and PCA was computed using the correlation matrix. The analyses were run in R 4.3.2 [45] using the *FactoMineR* 2.11 package to compute the PCA. The *factoextra* 1.0.7 package was used for visualisation, and graphics were created with *ggplot2* 3.5.1.

## 3. Results

### 3.1. Maturity Groups of Individuals

**Figure 3:**
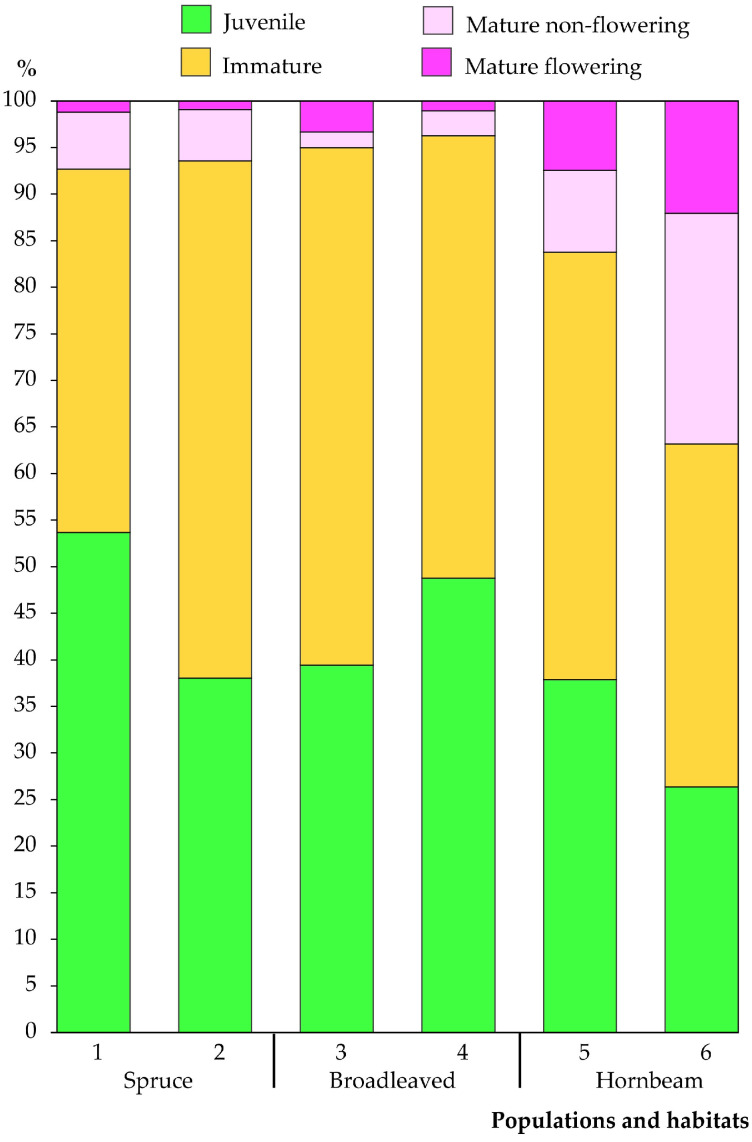
The proportion (in percent) of *Cardamine bulbifera* individuals classified by maturity group in the studied populations. Populations: Kaukinė (1), Pašuliai (2), Šešuva (3), Sutartiškės (4), Kalesninkai (5) and Vidzgiris (6).

A total of 2852 individuals were recorded and analysed in 120 sampling plots across the six *C. bulbifera* populations studied. Of the individuals analysed, 1,181 were classified as juvenile (41.4%), 1,362 as immature (47.8%), 194 as non-flowering mature (6.8%) and 115 as flowering mature (4.0%). Significant differences in the number and proportion of individuals by maturity group were found between the populations. The Vidzgiris population had the smallest number of juvenile individuals (83), while the Šešuva population had the largest (283). In terms of proportions (Figure 3), the largest percentage of juvenile individuals was found in the Kaukinė population (53.7%), and the smallest in the Vidzgiris population (26.3%).

The smallest number of immature individuals was found in the Vidzgiris population (116), and the largest was in the Šešuva population (399). In terms of proportion (Figure 3), immature plants comprised the largest part of individuals in the Šešuva population (55.6%) and the smallest part in the Vidzgiris population (36.8%). The proportion of young individuals (juvenile and immature) in the pooled sampled populations comprised 89.2% of all individuals.

The proportion of mature individuals in all sampled populations was relatively small, comprising 10.8% of all analysed individuals. In all studied populations except the Šešuva population, non-flowering mature individuals were more abundant than flowering mature individuals. The smallest number of non-flowering mature individuals was found in the Šešuva population (12), and the largest in the Vidzgiris population (78). In the pooled data set of all populations studied, non-flowering mature individuals accounted for 6.8%. However, the percentage of non-flowering mature individuals varied significantly between populations, ranging from 1.7% in the Šešuva population to 24.8% in the Vidzgiris population. Flowering mature individuals accounted for 4.0% of all populations, with their numbers in individual populations ranging from 3 in Pašuliai to 39 in Kalesninkai (or 0.9% and 7.5%, respectively; Figure 3). The Vidzgiris population demonstrated a significantly higher percentage of flowering mature individuals (12.1%), although the absolute number was almost equal to that of the Kalesninkai population (38 and 39 individuals, respectively).

An analysis of the proportions of different maturity groups of *C. bulbifera* individuals according to habitat type revealed significant differences between hornbeam forest and other habitats. The largest proportion of young individuals (combining juvenile and immature) was found in broadleaved forest habitats (95.5%), with a slightly smaller proportion found in spruce forest habitats (93.1%). A significantly smaller proportion of young individuals was found in hornbeam forest habitats (76.0%). The proportion of mature individuals (mature non-flowering and mature flowering combined) was the largest in hornbeam forest (24.0%), while in spruce (6.9%) and broadleaved forest (4.5%) habitats, their proportion was significantly lower.

### 3.2. Density of Individuals

Significant differences in density of individuals of all maturity groups were found among the six *C. bulbifera* populations studied (H = 31.5, *p* < 0.001). The highest density of individuals (Table 3) was found in the Šešuva population in a broadleaved forest (35.9 ± 14.3 individuals/m^2^), while the lowest density was found in the Vidzgiris population in a hornbeam habitat (15.8 ± 8.3 individuals/m^2^).

The density of individuals in different maturity groups also varied significantly across populations. The highest density of juvenile individuals was found in the Šešuva population, while the lowest density was found in the Vidzgiris population (Table 3). The highest density of immature individuals was found in the Šešuva population, where their density was significantly higher than in all the other studied populations (Table 3). As with juvenile individuals, the lowest density of immature individuals was found in the Vidzgiris population.

The highest density of mature non-flowering individuals was found in the Vidzgiris population, while the lowest density was recorded in the Šešuva population. In all studied populations except Šešuva, the density of mature flowering individuals was lower than that of mature non-flowering individuals (Table 3). The highest density of flowering individuals was found in the Kalesninkai population and the lowest in the Kaukinė population (Table 3).

Analysis of the density of all *C. bulbifera* individuals, as well as individuals of the studied maturity groups, considering the type of habitat revealed important patterns in the distribution of individuals. Significant differences in the density of individuals of all maturity groups combined were found among the studied habitats (H = 20.3, *p* < 0.001). The total mean density of individuals in broadleaved forest habitats (31.9 ± 14.0 individuals/m^2^) was significantly higher than in spruce (18.4 ± 14.7 individuals/m^2^) and hornbeam (20.9 ± 10.0 individuals/m^2^) forest habitats (*p* < 0.001). However, there were no significant differences in individual density between hornbeam and spruce forest habitats (z = 1.22, *p* = 0.223).

**Figure 4:**
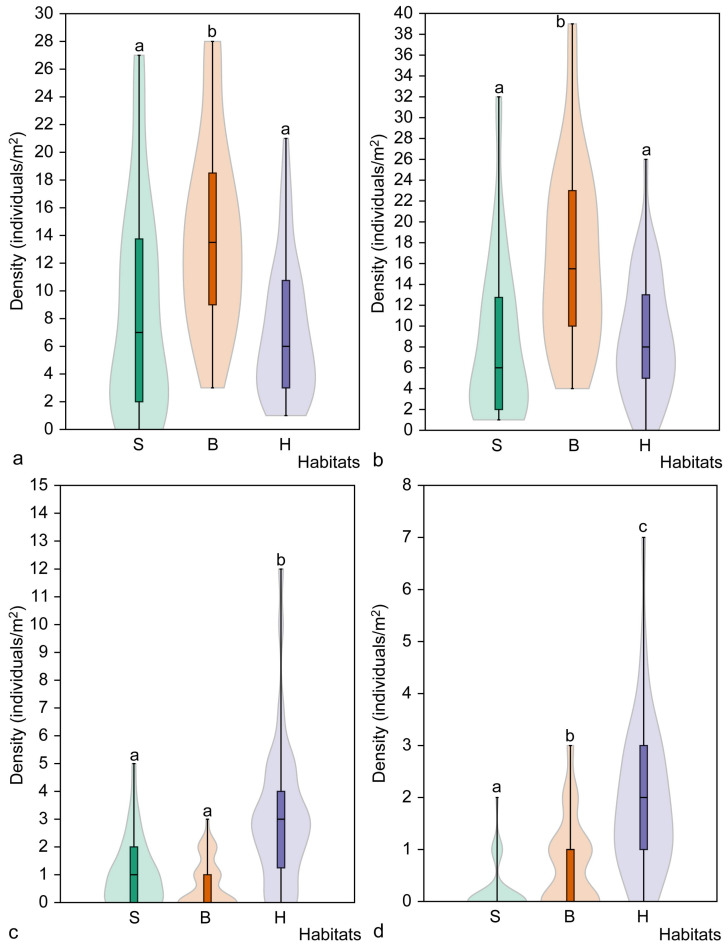
The mean density of juvenile (**a**), immature (**b**), non-flowering mature (**c**) and flowering mature (**d**) *Cardamine bulbifera* individuals in the studied habitat types. Different letters above the violin plots indicate significant differences (*p* < 0.05) between habitat types according to the results of Dunn’s post hoc test. Habitats: S, spruce forest; B, broadleaved forest; H, hornbeam forest.

The mean density of juvenile individuals in broadleaved forest habitats (13.9 ± 6.8 individuals/m^2^) was significantly higher than in spruce (z = 3.68, *p* < 0.001) and hornbeam forest (z = 4.27, *p* < 0.001) habitats. The same pattern was observed when analysing the density of immature individuals (Figure 4). The highest density of immature individuals was found in broadleaved habitats (16.6 ± 8.9 individuals/m^2^) and was significantly higher than in spruce (8.5 ± 7.8 individuals/m^2^) and hornbeam (8.9 ± 5.9 individuals/m^2^) forest habitats (*p* < 0.001). However, there were no significant differences in the density of immature individuals in spruce and hornbeam habitats (z = 0.64, *p* = 0.522).

The highest density of mature, non-flowering individuals was recorded in hornbeam habitats (3.1 ± 2.6 individuals per m^2^), which was significantly higher than in both spruce (z = 4.25, *p* < 0.001) and broadleaved forest (z = 5.50, *p* < 0.001) habitats (Figure 4). However, no differences were observed in the density of mature, non-flowering individuals between spruce and broadleaved forest habitats (z = 1.25, *p* = 0.211). Analysing the density of mature flowering individuals by habitat type revealed it to be lowest in spruce habitats (0.2 ± 0.5 individuals per m^2^) and highest in hornbeam forest habitats (1.9 ± 1.5 individuals per m^2^). Significant differences were found among all habitat types (H = 40.05, *p* < 0.001). The smallest difference in the number of mature flowering individuals was found between the spruce and broadleaved forest habitats (z = 2.77, *p* = 0.005).

### 3.3. Effect of Populations and Habitats on Plant Trait Variation

Analysis of the height of juvenile *C. bulbifera* individuals in the study populations showed that the differences were small, with height ranging from 3 cm to 9 cm (see Table 4). However, juveniles in the Pašuliai and Šešuva populations were significantly taller than those in the other four populations (*p* < 0.01), and a significant difference was observed between these two populations (*p* < 0.01). The mean height of juvenile individuals (*n* = 1181) across all studied populations was 5.7 ± 1.4 cm (Table 4).

The height of immature individuals in the studied populations ranged from 5 cm to 28 cm, with greater differences observed between populations than among juvenile individuals. As with the juvenile individuals, the tallest immature individuals were found in the Pašuliai population, where they were significantly taller (*p* < 0.05) than in all other populations. The mean height of immature individuals (*n* = 1276) in all populations studied was 15.8 ± 4.0 cm (Table 4).

**Figure 5:**
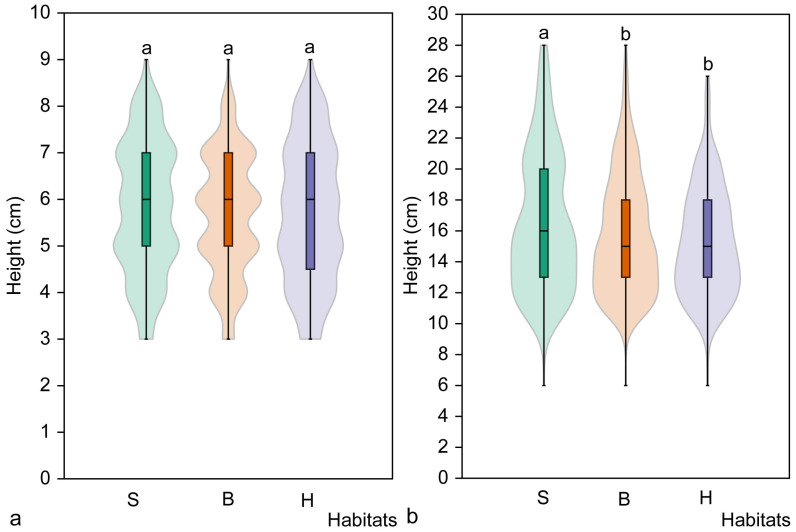
Mean height of juvenile (**a**) and immature (**b**) *Cardamine bulbifera* individuals in studied habitats. Different letters above the violin plots indicate significant differences (*p* < 0.05) between habitat types, as determined by the Mann–Whitney pairwise comparison. Habitats: S, spruce forest; B, broadleaved forest; H, hornbeam forest.

Analysis of the height of juvenile individuals in different habitat types revealed no significant differences (H = 2.09, *p* = 0.335). Pairwise comparison of habitat types also revealed no significant differences (Figure 5). Different results were obtained when comparing the height of immature individuals. Significant differences were found among populations (H = 11.05, *p* = 0.004), with immature individuals in spruce habitats being significantly taller than those in broadleaved (*p* = 0.014) or hornbeam (*p* = 0.001) habitats (Figure 5).

The height of non-flowering, mature *C. bulbifera* individuals in the studied populations ranged from 14 cm to 56 cm, with a mean height of 38.1 ± 7.2 cm (Table 5). Significant differences in plant height were found among the studied populations (H = 11.42, *p* = 0.043). Non-flowering mature individuals in the Sutartiškės population were the shortest, differing significantly (*p* < 0.05) from all other populations except the Šešuva (*p* = 0.217) and Pašuliai (*p* = 0.173) populations (Table 5).

The length of the stem section of non-flowering mature individuals with bulbils in the leaf axils ranged from 5 cm to 37 cm across the studied populations. The mean length of the stem section with bulbils of all analysed individuals in this group (*n* = 194) was 20.1 ± 6.6 cm (Table 5). Although significant differences were found among all the populations studied (H = 14.03; *p* = 0.015), a pairwise comparison revealed that the Sutartiškės population was the only one that differed significantly (*p* < 0.05) from the others. The stem section with axillary bulbils was significantly shorter than in the other populations (Table 5).

Non-flowering mature individuals of *C. bulbifera* produced 2 to 18 axillary bulbils in the study year, and there was a significant difference in the number of bulbils produced among populations (H = 65.53, *p* < 0.001). The mean number of bulbils produced by all non-flowering mature individuals (*n* = 194) was 9.1 ± 3.6 (Table 5). Plants in this maturity group in the Kalesninkai population produced the lowest number of bulbils, differing significantly (*p* < 0.001) from all other populations (Table 5).

**Figure 6:**
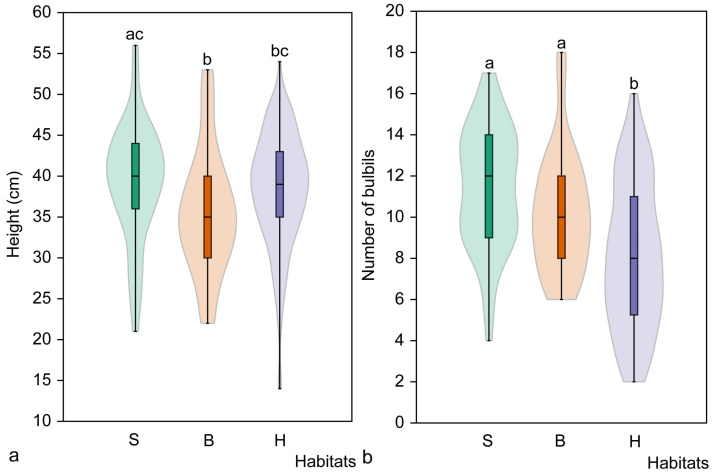
Mean height (**a**) and mean number of bulbils (**b**) of non-flowering mature *Cardamine bulbifera* individuals in studied habitats. Different letters above the violin plots indicate significant differences (*p* < 0.05) between habitat types, as determined by the Mann–Whitney pairwise comparison. Habitats: S, spruce forest; B, broadleaved forest; H, hornbeam forest.

The height of non-flowering mature *C. bulbifera* individuals differed significantly among the studied habitat types (H = 6.30, *p* = 0.042). Significant differences were found between spruce and broadleaved forest habitats, and between broadleaved and hornbeam forest habitats (Figure 6). However, no significant differences in plant height were found between spruce and hornbeam habitats (*p* = 0.511). The tallest plants (39.2 ± 7.2 cm) were found in spruce habitats, while those in broadleaved forest habitats were significantly shorter (35.7 ± 7.7 cm).

The length of the stem section with axillary bulbils of non-flowering mature *C. bulbifera* individuals also differed significantly among the studied habitats (H = 8.31, *p* = 0.015). Significant differences were found between spruce and broadleaved habitats, as well as between broadleaved and hornbeam forest habitats. The longest stem sections with axillary bulbils were observed in spruce habitats (21.1 ± 7.3 cm), while the shortest were found in broadleaved forest habitats (17.3 ± 7.4 cm). No significant differences in plant height were found between spruce and hornbeam habitats only (*p* = 0.541).

The number of axillary bulbils on non-flowering mature *C. bulbifera* individuals also differed significantly (H = 29.08, *p* < 0.001) between habitats. Significant differences in the number of axillary bulbils were found between hornbeam and broadleaved forest habitats, as well as between hornbeam and spruce habitats (Figure 6). The smallest mean number of bulbils produced was in the hornbeam habitat (8.1 ± 3.5), while the largest mean number was in the spruce habitat (11.4 ± 2.9). No significant differences in the number of bulbils were found between spruce and broadleaved forest habitats (*p* = 0.065).

The mean height of flowering mature individuals in all the studied populations was 51.8 ± 9.2 cm, ranging from 22 cm to 75 cm (Table 6). No significant difference in height was found between flowering mature individuals in all studied populations (H = 7.94, *p* = 0.159). However, flowering mature individuals of the Šešuva population were significantly shorter than those of the Kalesninkai population (*p* = 0.005). The tallest flowering mature individuals were recorded in the Kalesninkai population (Table 6).

The mean length of the stem section with axillary bulbils of flowering, mature *C. bulbifera* individuals in all populations was 30.1 ± 6.7 cm, ranging from 12 cm to 44 cm (Table 6). Significant differences in the length of the stem section with axillary bulbils were found among the studied populations (H = 11.76, *p* = 0.038). However, a pairwise comparison revealed significant differences between the Kalesninkai and Šešuva populations, as well as between the Kalesninkai and Vidzgiris populations. The longest stem sections with axillary bulbils were recorded in the Kalesninkai population, in which individuals of this maturity group were the tallest (Table 6).

In the studied year, flowering mature *C. bulbifera* individuals produced between 2 and 21 axillary bulbils. A significant difference in the number of bulbils produced was found among populations (H = 55.57, *p* < 0.001). The mean number of bulbils produced by all flowering mature individuals (*n* = 115) was 12.4 ± 4.9. As with non-flowering mature individuals, flowering mature individuals in the Kalesninkai population produced the lowest number of bulbils, differing significantly (*p* < 0.01) from all other populations (Table 6).

The inflorescence of flowering mature *C. bulbifera* individuals was generally short (2.5 ± 1.2 cm), ranging from 1 cm to 7 cm. A significant difference in inflorescence length was observed among all populations (H = 31.01, *p* < 0.001). The shortest inflorescences were found in the Vidzgiris population, whereas the longest were found in the Kalesninkai population. Inflorescences in this population were significantly longer than those recorded in all the other populations studied (Table 7).

*Cardamine bulbifera* individuals generally produced a small number of flowers (4.1 ± 2.1), ranging from 1 to 11 flowers per inflorescence. A significant difference in the number of flowers produced was found among all the studied populations (H = 18.98, *p* < 0.002). The Vidzgiris population had the smallest mean number of flowers, whereas the Kalesninkai population had the largest (Table 7).

**Figure 7:**
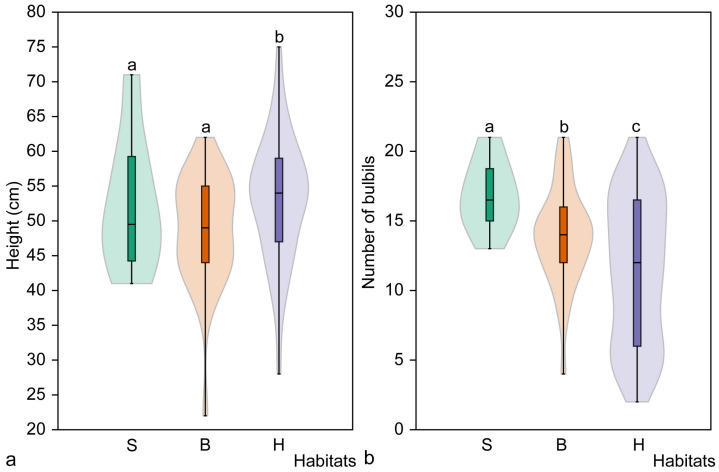
Mean height (**a**) and mean number of bulbils (**b**) of mature flowering *Cardamine bulbifera* individuals in studied habitats. Different letters above the violin plots indicate significant differences (*p* < 0.05) between habitat types, as determined by the Mann–Whitney pairwise comparison. Habitats: S, spruce forest; B, broadleaved forest; H, hornbeam forest.

Analysis of the height of mature flowering *C. bulbifera* individuals showed no significant differences among habitat types (H = 5.45, *p* = 0.065), but plants in the hornbeam habitat were significantly (*p* = 0.019) taller than those in the broadleaved forest habitat (Figure 7). No significant differences were found in the length of the stem section with axillary bulbils of mature flowering *C. bulbifera* individuals in the studied habitats (H = 4.60, *p* = 0.099), nor between pairs of habitats (*p* > 0.05).

Different regularities were found when analysing the number of axillary bulbils on flowering mature *C. bulbifera* individuals (Figure 7). The number of bulbils differed significantly among all habitats (H = 11.01, *p* < 0.004) and between all pairs of habitats (*p* < 0.05). The largest mean number of axillary bulbils was recorded in spruce habitat (16.7 ± 2.5), whereas the lowest mean number was found in hornbeam habitat (11.4 ± 5.2).

**Figure 8:**
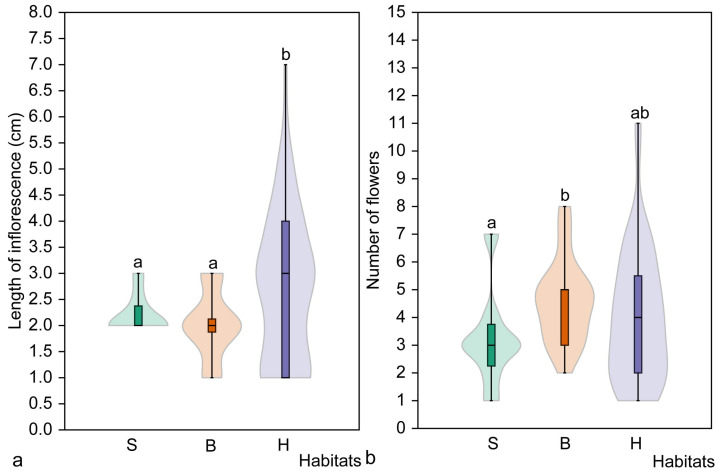
Mean length of inflorescence (**a**) and mean number of flowers (**b**) of mature flowering *Cardamine bulbifera* individuals in studied habitats. Different letters above the violin plots indicate significant differences (*p* < 0.05) between habitat types, as determined by the Mann–Whitney pairwise comparison. Habitats: S, spruce forest; B, broadleaved forest; H, hornbeam forest.

Significant differences in inflorescence length were found among all habitat types (H = 5.7, *p* = 0.047), but a pairwise comparison revealed a significant difference (*p* = 0.193) only between hornbeam and broadleaved forest habitats (Figure 8). The mean length of inflorescence was smallest in the broadleaved forest habitat (2.0 ± 0.6 cm). However, the opposite results were obtained when the number of flowers was compared. No significant differences were found among all habitat types (H = 5.12, *p* = 0.073), and the largest number of flowers was found in the broadleaved forest habitat (4.6 ± 1.5). A significant difference (*p* = 0.020) in the number of flowers was found between flowering individuals in spruce and broadleaved forest habitats (Figure 8).

### 3.4. The Effect of Species Diversity on Population Structure

The number of plant species in the sampling plots of all the *C. bulbifera* populations studied differed significantly (H = 62.48, *p* < 0.001). The number of plant species recorded in individual sampling plots ranged from 5 to 23. The highest mean number of species was found in the Kaukinė population (17.7 ± 3.2), while the lowest was recorded in the Kalesninkai population (10.0 ± 3.1). The similarity in species diversity between the populations studied was relatively low, ranging from 16% to 50%. The highest similarity of species was found between the Kaukinė and Vidzgiris populations (J = 0.50) and between the Sutartiškės and Kaukinė populations (J = 0.47), whereas the lowest species similarity was found between the Pašuliai and Sutartiškės populations (J = 0.16). Except for *Cardamine bulbifera*, only two species, *Anemone nemorosa* and *Lamium galeobdolon*, were recorded in all studied populations with high frequency (100% and 87.5% of all sampling plots, respectively). Only 3 of the 31 total plant species registered in the sampling plots were recorded in all the studied populations: *Anemone nemorosa*, *Cardamine bulbifera* and *Lamium galeobdolon*. Furthermore, *Anemone nemorosa* and *Cardamine bulbifera* were found in all sampling plots (frequency 100%) of all studied sites and habitat types. The frequency of the other species varied significantly between populations and habitat types.

Analysis of the number of species in the sampling plots of the studied habitats revealed that diversity was highest in the broadleaved forest habitat (16.6 ± 2.5 species), similar in the spruce habitat (15.8 ± 3.5 species) and lowest in the hornbeam habitat (11.2 ± 2.8 species). There was a significant difference in the number of plant species recorded in the sampling plots of the studied habitats (H = 49.22, *p* < 0.001). When the habitats were compared in pairs, it was found that the hornbeam habitat had the lowest mean number of species and differed significantly from the broadleaved and spruce forest habitats (*p* < 0.001).

Linear correlation analysis revealed weak though reliable negative relationships between the number of species in a plot and both the number of non-flowering mature individuals (r = −0.29, *p* = 0.002) and the number of flowering mature individuals (r = −0.36, *p* < 0.001). A weak positive correlation (r = 0.25, *p* = 0.006) was observed between the number of species in a plot and the number of juvenile individuals. No reliable correlations were found between the cover of plant species and the total number of *C. bulbifera* individuals, nor between most maturity groups. However, a weak negative correlation was found between the number of flowering mature individuals and the total herb cover (r = −0.27; *p* = 0.003).

### 3.5. The Effect of Habitat Characteristics on Population Structure

The mean plant height in the sampling plots of all the studied populations was 16.9 ± 4.2 cm, ranging from 12.8 ± 2.0 cm in the Pašuliai population to 22.8 ± 5.2 cm in the Vidzgiris population. In individual populations, plant debris covered between 41.7% (Vidzgiris) and 81.6% (Sutartiškės) of the soil surface. The percentage of bare soil was generally low in most populations (ranging from 1.4% to 2.9%), except in hornbeam habitats where it constituted 45.5% in the Kalesninkai population and 52.5% in the Vidzgiris population. The cover of bryophytes was low in all populations, ranging from 0.8% in the Kalesninkai population to 6.4% in the Pašuliai population. Herbaceous plants covered between 11.2% (Kalesninkai) and 66.2% (Pašuliai) of the ground surface.

**Figure 9:**
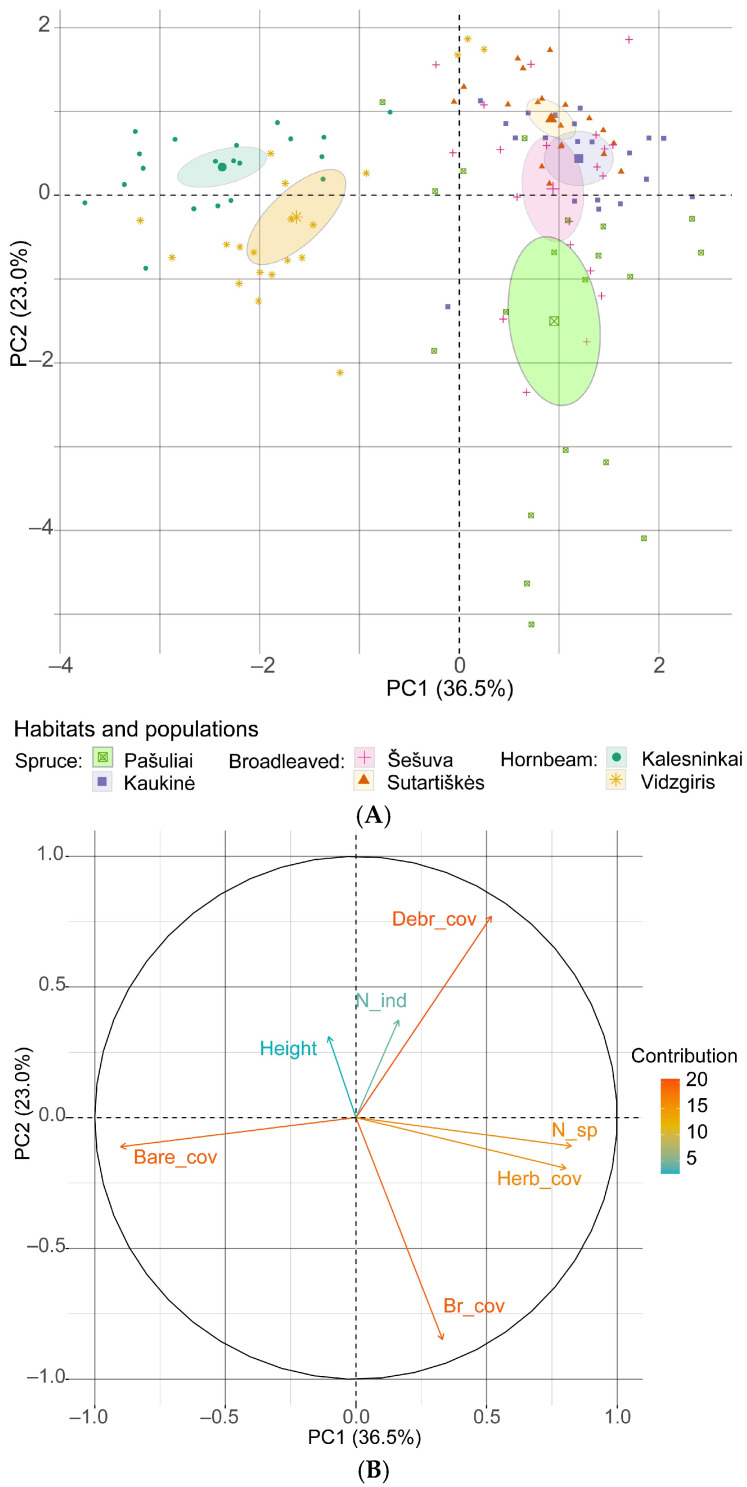
Principal component analysis of the relationship between the total number of *Cardamine bulbifera* individuals and habitat variables: (**A**) scores for the studied populations with 95% confidence limits (ellipses); (**B**) loading plot of the variables contributing to PC1 and PC2. Abbreviations: Bare_cov: cover of bare soil; Br_cov: cover of bryophytes; Debr_cover: cover of plant debris; Height: mean height of herbaceous plants in the sampling plot; Herb_cov: cover of herbaceous plants; N_sp: number of plant species; N_ind: number of *C. bulbifera* individuals.

An analysis of the principal components, which included the most significant habitat and community characteristics, revealed that the first component accounted for 36.5% (eigenvalue λ = 2.55) of the variation in the total number of *C. bulbifera* individuals, while the second component accounted for 23.0% (λ = 1.61). Together, the first two principal components explain 59.5% of the variation (Figure 9). The strongest negative loadings were associated with the area of bare soil (−0.901) in the first component and the number of bryophyte species (−0.848) in the second component. The strongest positive loadings in the first principal component were associated with the number of plant species (0.824) and total herb cover (0.803).

**Figure 10:**
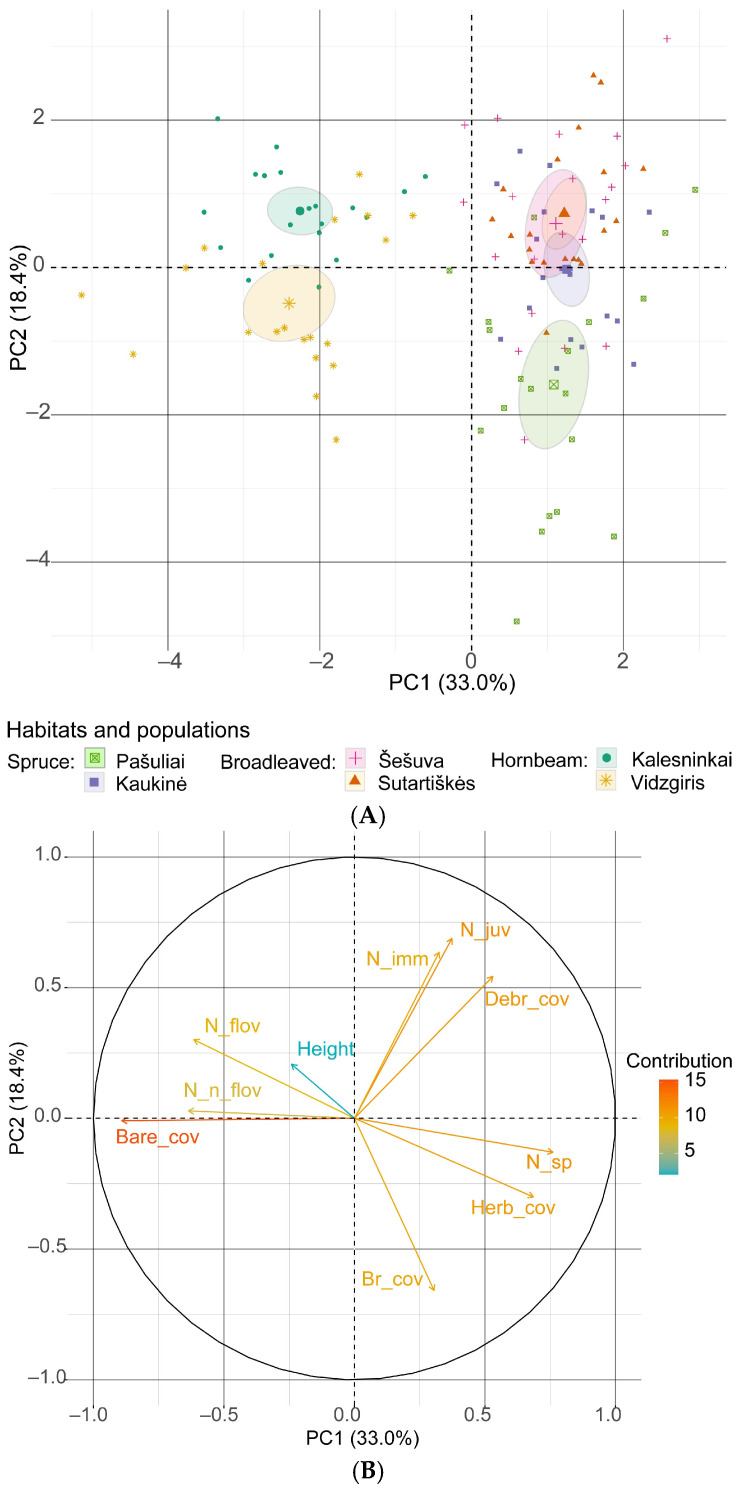
Principal component analysis of the relationship between the total number of *Cardamine bulbifera* individuals and habitat variables: (**A**) scores for the studied populations with 95% confidence limits (ellipses); (**B**) loading plot of the variables contributing to PC1 and PC2. Abbreviations: Bare_cov: cover of bare soil; Br_cov: cover of bryophytes; Debr_cover: cover of plant debris; Height: mean height of herbaceous plants in the sampling plot; Herb_cov: cover of herbaceous plants; N_sp: number of plant species; N_juv: number of juvenile *C. bulbifera* individuals; N_imm: number of immature individuals; N_flov: number of flowering mature individuals; N_n_flov: number of non-flowering mature individuals.

An analysis of the effect of environmental factors on the four maturity groups of *C. bulbifera* individuals using principal components revealed that the first principal component explains 33.0% of the variation (λ = 3.30), and the second component explains 18.4% of the variation (λ = 1.84). The first two principal components combined account for 51.4% of the variation (Figure 10). The strongest negative loadings were associated with area of bare soil in the first component (−0.891) and number of bryophyte species in the second component (−0.657). The strongest positive loadings in the first principal component were associated with the number of plant species (0.760) and total herb cover (0.685).

The results of the principal component analysis revealed that bare soil had the strongest negative effect on the density of *C. bulbifera* individuals. However, herb cover, cover of plant debris and species richness had a positive effect on the abundance of young and immature individuals, but a negative effect on mature individuals. In both analyses, the contribution of the mean height of herbaceous plants in the community to the abundance of *C. bulbifera* individuals was low.

## 4. Discussion

### 4.1. Maturity Groups of Individuals

The results of our study showed that flowering individuals constitute a very small proportion of all individuals in populations (4.0% of the total of 2852 recorded individuals), and neither their total number nor their density per unit area accurately reflects the overall density and abundance of individuals in the population (Table 3). A large proportion of juvenile and immature individuals remain unnoticed during surveys, giving the false impression that the population is small or under threat [31,39,46]. The small number of flowering individuals is probably an individual characteristic of *C. bulbifera*, given that the plant reproduces almost exclusively or completely asexually by axillary bulbils [34,35].

The ratio of young to mature individuals is often used to assess the status of plant populations [47]. According to this criterion, it is possible to determine whether a population is dynamic, normal or regressive [13,47,48,49,50]. Analysis of *C. bulbifera* populations showed that, based on the principle that an abundance of young individuals (including juveniles and immature plants) indicates a dynamic state, all populations studied should be classified as dynamic. However, since we could not find any published information on the distribution of *C. bulbifera* individuals in populations according to maturity group in other areas of its range and all existing information relates only to the abundance of flowering individuals, doubts remain as to whether this assessment truly reflects the state of the population. Previous studies have shown that the abundance of non-flowering individuals in some plant populations does not indicate good condition, and conversely, a large proportion of flowering individuals does not indicate population degradation [50,51,52]. It has been found that a high proportion of non-flowering individuals in *Cephalanthera longifolia* populations does not indicate a dynamic state but rather unfavourable habitat conditions, most often a lack of sunlight [50].

According to our research, a portion of *Cardamine bulbifera* individuals are non-flowering mature plants. These plants have all the characteristics of flowering mature individuals, but do not produce flowers. This group of individuals was not previously distinguished, but we believe that it should be considered a subgroup of mature individuals (they produce stems and axillary bulbils, but do not form flowers). It is possible that such plants represent a developmental stage at which they lose the ability to reproduce sexually and do not invest energy in flower formation [53]. However, it is more likely that non-flowering mature individuals represent an intermediate stage between immature and flowering mature individuals [54]. To determine the exact causes of this phenomenon, however, long-term stationary population studies in the wild or experimental studies under artificial, controlled conditions are needed.

### 4.2. Density of Individuals

The study of the density of *C. bulbifera* individuals revealed significant and reliable differences between populations. In our opinion, these differences were not caused by random factors, natural variations, or fluctuations in population status, but rather by a combination of habitat conditions. The highest total density of individuals of all maturity groups was found in the habitat of broadleaved forest, particularly in the Šešuva population, which occurred in an old natural forest (Table 1). The study area was dominated by over 200-year-old *Quercus robur* trees. Additionally, the habitat contained dead trees, creating a mosaic of open areas and gaps under the tree canopy characteristic of biologically mature trees in old forests [55]. The significantly higher density of *Cardamine bulbifera* individuals in these forests, compared to populations in spruce and hornbeam habitats, confirms that this species is characteristic of old forests [56,57]. In spruce and hornbeam habitats where the forest stand was more uniform (with trees of almost the same age and a canopy with few gaps), and where good light conditions only occurred in early spring, the density of *C. bulbifera* individuals was significantly lower. Therefore, it can be assumed that light conditions, dependent on the vertical structure of stand-forming trees, are the most significant environmental factor influencing population density [58,59].

Analysis of individuals from different maturity groups revealed that their density also varied greatly depending on habitat type. The highest density of juvenile individuals was found in broadleaved forest habitats, while the lowest density was found in hornbeam forest habitats. Recruitment is much more intense in broadleaved forest habitats than in other types, particularly hornbeam forests (Table 2). It should be noted that no significant relationships were found between the density of young individuals (juvenile and immature) and the density of mature individuals in the study plots (Table 3). There was also no correlation between the number of bulbils produced by mature individuals and the density of young individuals. This suggests that young individuals reach maturity within a few years, thus explaining the lack of correlation between their density and the production of bulbils [34]. There are no precise data on how long it takes individuals grown from bulbils to reach the immature or mature stages. To determine this timeframe, long-term field studies or experimental studies under artificial conditions that imitate the natural forest habitat are required.

Interestingly, in hornbeam forest habitats, particularly in the Vidzgiris population, the density and proportion of mature individuals were significantly higher than in other habitats, whereas the density of juvenile and immature individuals in these habitats was significantly lower. This raises the question of what causes the difference in density of mature individuals, and whether this is because hornbeam forest habitats have more favourable light conditions, particularly in spring, or because the renewal of *C. bulbifera* populations in hornbeam habitats is much slower despite relatively high bulbils production [60,61]. It is possible that recruitment in hornbeam habitats is limited by habitat conditions or other factors. Since a relatively large part of the soil surface in these habitats was bare, recruitment may be inhibited by water erosion: fallen bulbils are washed away and die [62]. Furthermore, it is possible that most bulbils on bare soil are destroyed by herbivores (probably invertebrates), whereas those that fall among plant debris are more likely to survive as they are harder for herbivores to find [63]. The higher density of mature individuals in hornbeam habitats cannot be explained by population age, as all populations studied have existed for over two decades. Therefore, none of the populations can be considered young, despite the higher density of young individuals.

The questions raised by this study can only be answered through long-term research in permanent sampling plots. Determining long-term bulbil production, recruitment intensity and the development of young individuals grown from bulbils is only possible through long-term studies. The results of this study suggest that old broadleaved forest habitats which have remained undisturbed by humans for many years provide the most favourable conditions for recruitment, and these habitats should therefore be considered particularly important for the conservation of this species.

### 4.3. Effect of Populations and Habitats on Plant Trait Variation

Analysis of the morphological characteristics of *C. bulbifera* individuals showed that the traits of juvenile and immature individuals were highly consistent across all studied populations, with only minor differences observed between populations and habitats (Table 4). Slightly greater differences were found only in the height of immature individuals. The greater variation in height among immature individuals is likely to be due to differences in absolute age within this maturity group. However, this can only be confirmed or disproved by a detailed study of individual development and the relationship between morphological traits and absolute age [64,65]. Therefore, it can be concluded that the conditions for the development of young individuals were similarly favourable in all populations and habitats studied, despite their differences. As *C. bulbifera* is a typical forest plant, its young individuals can tolerate light deficiency or competition with other plants [58,66].

Significantly greater differences were found in the traits of mature individuals at both the population and habitat levels (Table 5). The tallest non-flowering and flowering mature individuals were found in the spruce forest habitat, while the shortest were found in the broadleaved forest habitat. The same pattern was observed when analysing the length of the stem section with bulbils. This suggests that the amount of light in the habitat may have influenced plant height [58,59]. Light availability was lowest in spruce habitats, where tree cover was at its maximum of 70%. In the more illuminated broadleaved forest habitats, the plants were shorter. However, non-flowering and flowering mature individuals in the broadleaved forest habitats produced more bulbils than in the spruce and hornbeam habitats. Therefore, it can be concluded that plant height is not a trait that determines the production of bulbils.

Interestingly, the opposite pattern emerged when inflorescence length and the number of flowers per inflorescence were assessed. The longest inflorescences were found in hornbeam habitats, whereas spruce habitats had significantly more flowers than broadleaf forest habitats (Table 6). This phenomenon cannot be explained by light availability alone. It is possible that different microevolutionary processes are taking place in different, relatively isolated populations [35,67]. In spruce and broadleaf forests, *C. bulbifera* invests less in inflorescence formation and flowering. However, the flowering does not contribute to reproduction as the plants do not ripen their seeds. Our observations showed that in the two hornbeam habitats studied, the plants set fruit and matured seeds, indicating that different microevolutionary processes occur in populations established in these habitats. Nevertheless, the influence of sexual reproduction on population renewal remains unclear [34]. To assess the importance of sexual reproduction for the stability of *C. bulbifera* populations in hornbeam habitats, studies focusing on fruit set and seed viability are required.

Analysis of *C. bulbifera* trait variation between populations and habitats indicates that undisturbed, old, broadleaved forest habitats with natural succession are most favourable for its growth and most important for its conservation. Stable and abundant populations of this species are supported by species-rich spruce forests and hornbeam habitats.

### 4.4. Relationships Between Species Diversity and Population Structure

The negative relationship between higher species diversity and the number of mature *C. bulbifera* individuals in the sampling plot, as well as the negative correlation between the number of mature individuals and plant cover, suggests that *C. bulbifera* may be sensitive to competition. With greater species diversity, competition within the community for space, light and nutrients increases [67,68]. Competition for light may have the greatest impact, given that *C. bulbifera* has an early growing season and flowers before the trees in the stand fully expand their leaves, therefore allowing more sunlight to reach the ground [69]. However, when the diversity or abundance of rapidly growing spring plants increases, so does competition between them, which may suppress the development of young individuals [68]. It is possible that immature *C. bulbifera* individuals do not accumulate enough energy to reach maturity in plots with greater species diversity and herbaceous plant cover. This could explain why the proportion of immature individuals is much higher in habitats with dense herb cover, such as spruce and broadleaved forests, than in hornbeam forests with much sparser herb cover.

### 4.5. The Effect of Habitat Characteristics on Population Structure

An analysis of habitat and community characteristics and their effect on the abundance of *C. bulbifera* individuals revealed that conditions in both spruce and broadleaved forests were much more similar than in hornbeam forests. Despite variations in the mean height of plants within communities between populations, this factor had little effect on the abundance of *C. bulbifera* individuals. Differences in mean herb layer height were mainly determined by the diversity and abundance of dominant species [59]. The most pronounced differences from the other populations studied were found in the hornbeam habitat of the Vidzgiris population. The mean plant height could not have been influenced by the timing of the studies, as they were conducted in all populations within a 4-day timeframe.

The area of bare soil within the community significantly affected the abundance of *C. bulbifera* individuals. In addition to the aforementioned possible negative impact of bare soil (e.g., bulbils being accessible to herbivores or being carried away from the habitat), bare soil and increased erosion may affect the viability of the rhizomes of individuals of all age groups, particularly increasing the mortality of juvenile individuals grown from bulbils [70]. In populations with a thick layer of plant debris, the soil is protected from erosion and the bulbils are protected from herbivores, creating favourable microhabitat conditions that allow individuals grown from bulbils to establish more easily in the soil [71]. The soil under the layer of debris retains moisture for a longer period of time, meaning that young plants experience less stress from environmental factors such as frost and drought [72]. In some cases, bare soil in a community can also have a positive effect, particularly on population recruitment from seeds. Soil disturbance can promote the germination of seeds and the renewal of populations of short-lived species with a ruderal strategy or even foster the introduction of non-native species [73,74]. However, in the case of the long-lived, rhizomatous *C. bulbifera*, soil disturbance has the opposite effect on population recruitment.

The abundance of young *C. bulbifera* individuals, as well as the total number of individuals in the population, correlated positively with the number of plant species and the cover of the herb layer. Both species diversity and the cover of the herb layer are indicators of habitat stability. Therefore, it can be assumed that *C. bulbifera* is sensitive to habitat disturbances, and that favourable conditions occur in stable habitats with a certain degree of dynamism and gradual change. The results obtained confirm the assumption that old, biologically mature forest stands are important for protecting *C. bulbifera* populations and all biological diversity in forests [57,75].

The results of this study could help to organise the protection of *C. bulbifera* and other rare forest species, as well as their habitats. Old forests, in which all processes occur naturally with minimal direct disturbance, should be prioritised for conservation [76,77]. Long-term studies in permanent study plots or under experimental conditions could answer some remaining questions regarding the development of *C. bulbifera* and its sexual reproduction. Much larger-scale studies, including several regions within the *C. bulbifera* range, are needed to determine how proportions of individuals of different maturity groups within populations should be interpreted. Clearly, the current understanding of the population structure of certain ecological and biological plant groups is inadequate.

## 5. Conclusions

The density of *C. bulbifera* is significantly influenced by habitat structure. Old broadleaved forests support the highest population densities due to favourable light conditions and habitat complexity. In contrast, spruce and hornbeam forests, which have more uniform canopies and slightly less favourable microhabitats, support lower-density populations.

Recruitment of young individuals is most intense in broadleaved forest habitats, while hornbeam forests show high densities of mature plants but lower recruitment. This suggests that unfavourable soil conditions, such as bare soil and erosion, may inhibit the successful establishment of bulbils or lead to the death of juveniles.

The traits of young *C. bulbifera* individuals were consistent across habitats. However, mature plants showed trait variation, such as differences in height and reproductive structures, which are linked to the light regime in the habitat and possibly to local microevolutionary processes occurring in the populations.

A thick layer of plant debris and high plant diversity have a positive impact on the abundance and recruitment of *C. bulbifera*, highlighting its sensitivity to soil erosion and habitat disturbance. Stable, undisturbed habitats are essential for maintaining viable populations.

The study confirms that biologically mature broadleaved forests provide the most favourable conditions for the survival and reproduction of *C. bulbifera*. Conservation efforts should prioritise the protection of undisturbed old-growth forests to ensure the survival of this and other forest-dependent species.

The preservation of the natural composition of tree species and the heterogeneity of stand structure may contribute to the vitality and temporal stability of *C. bulbifera* populations, among other factors. Therefore, continuous cover forestry is an important approach to increasing the survival of forest understorey specialists, a consideration that will become increasingly relevant in the context of global climate change and intensified large-scale forest disturbances.

## Figures and Tables

**Table 1:** Location and habitat types of *Cardamine bulbifera* study sites.

Site	District	Habitat Type	Habitat Name in the Text
Šešuva	Kaišiadorys	9020 Fennoscandian hemiboreal natural old broadleaved deciduous forests	Broadleaved forest
Pašuliai	Kaišiadorys	9050 Fennoscandian herb-rich forests with *Picea abies*	Spruce forest
Sutartiškės	Kaišiadorys	9020 Fennoscandian hemiboreal natural old broadleaved deciduous forests	Broadleaved forest
Kaukinė	Kaišiadorys	9050 Fennoscandian herb-rich forests with *Picea abies*	Spruce forest
Kalesninkai	Alytus	9170 *Galio-Carpinetum* oak–hornbeam forests	Hornbeam forest
Vidzgiris	Alytus	9170 *Galio-Carpinetum* oak–hornbeam forests	Hornbeam forest

**Table 2:** Vegetation layer and soil surface cover (%) at *Cardamine bulbifera* study sites.

Site	Tree Layers			Shrubs	Herbs	Bryophytes	Plant Debris	Bare Soil
	Upper	Lower	Total					
Šešuva	30	30	40	60	60	10	70	3
Pašuliai	50	40	70	20	60	5	70	5
Sutartiškės	50	30	60	30	70	15	70	3
Kaukinė	40	50	70	40	80	5	80	2
Kalesninkai	70	30	70	10	40	1	60	40
Vidzgiris	40	65	70	40	60	1	30	50

**Table 3:** The mean density of individuals in the studied *Cardamine bulbifera* populations per 1 m^2^ (mean ± SD). Different letters in superscript across the same column indicate significant differences between populations according to the results of the Dunn’s post hoc test (*p* < 0.05).

Population	Forest Type	Individuals of All Groups	Juveniles	Immature	Mature Non-Flowering	Mature Flowering
Kaukinė	Spruce	20.5 ± 12.6 ^ade^	11.0 ± 7.7 ^aef^	8.0 ± 5.1 ^a^	1.3 ± 1.4 ^a^	0.2 ± 0.6 ^a^
Pašuliai	Spruce	16.3 ± 16.6 ^ad^	6.2 ± 7.0 ^b^	9.1 ± 9.8 ^ab^	0.9 ± 1.0 ^a^	0.2 ± 0.4 ^a^
Šešuva	Broadleaved	35.9 ± 14.3 ^b^	14.2 ± 6.2 ^ce^	19.9 ± 9.3 ^c^	0.6 ± 0.7 ^a^	1.2 ± 0.9 ^b^
Sutartiškės	Broadleaved	28.0 ± 12.9 ^bc^	13.7 ± 7.5 ^c^	13.3 ± 7.1 ^d^	0.8 ± 1.0 ^a^	0.3 ± 0.5 ^a^
Kalesninkai	Hornbeam	26.2 ± 8.8 ^ec^	9.9 ± 4.4 ^df^	12.0 ± 5.2 ^bd^	2.3 ± 1.6 ^b^	2.0 ± 1.5 ^b^
Vidzgiris	Hornbeam	15.8 ± 8.3 ^d^	4.2 ± 3.8 ^b^	5.8 ± 4.8 ^a^	3.9 ± 3.1 ^b^	1.9 ± 1.5 ^b^
Pooled data		23.8 ± 14.2	9.8 ± 7.2	11.3 ± 8.4	1.6 ± 2.0	1.0 ± 1.2

**Table 4:** The mean height (mean ± SD) and height range of juvenile and immature *Cardamine bulbifera* individuals in the studied populations. Different letters in superscript across the same column indicate significant differences between populations according to the Mann–Whitney test results (*p* < 0.05).

Population	Juveniles			Immature		
	Number of Individuals (*n*)	Plant Height (cm)	Range (Min–Max; cm)	Number of Individuals (*n*)	Plant Height (cm)	Range (Min–Max; cm)
Kaukinė	220	5.5 ± 1.9 ^a^	3–8	160	15.5 ± 3.8 ^ab^	10–28
Pašuliai	124	6.3 ± 1.7 ^b^	4–9	181	17.5 ± 4.8 ^d^	6–28
Šešuva	283	5.8 ± 1.9 ^c^	3–9	398	16.6 ± 4.1 ^c^	6–28
Sutartiškės	273	5.4 ± 1.4 ^a^	3–8	266	14.4 ± 3.0 ^ef^	10–24
Kalesninkai	198	5.7 ± 1.5 ^a^	3–9	155	14.9 ± 3.6 ^af^	10–25
Vidzgiris	83	5.7 ± 1.9 ^a^	3–9	116	15.7 ± 3.4 ^bc^	5–26
Pooled data	1181	5.7 ± 1.4	3–9	1276	15.8 ± 4.0	5–28

**Table 5:** The mean plant height, mean length of the stem section with axillary bulbils and mean number of bulbils of non-flowering mature *Cardamine bulbifera* individuals in the studied populations. Significant differences between populations according to the Mann–Whitney test results (*p* < 0.05) are indicated by different letters in superscript across the same column.

Population	Number of Individuals (*n*)	Plant Height (cm)	Range (Min–Max; cm)	Length of Stem Section with Bulbils (cm)	Range (Min–Max; cm)	Number of Bulbils	Range (Min–Max)
Kaukinė	25	41.0 ± 7.5 ^a^	21–56	21.1 ± 6.9 ^a^	7–37	11.6 ± 2.6 ^a^	6–16
Pašuliai	18	36.7 ± 6.2 ^b^	24–46	21.1 ± 8.2 ^a^	5–33	11.1 ± 3.3 ^ac^	4–17
Šešuva	15	37.5 ± 8.5 ^a^	22–53	21.1 ± 8.0 ^a^	10–30	10.6 ± 3.5 ^ac^	7–18
Sutartiškės	12	34.2 ± 6.9 ^c^	23–49	14.3 ± 5.5 ^b^	5–27	10.0 ± 2.4 ^ac^	6–13
Kalesninkai	46	38.5 ± 6.6 ^a^	15–49	20.3 ± 6.3 ^a^	5–34	5.6 ± 2.7 ^b^	2–13
Vidzgiris	78	38.3 ± 7.2 ^a^	14–54	20.4 ± 5.8 ^a^	6–32	9.6 ± 3.0 ^bc^	2–16
Pooled data	194	38.1 ± 7.2	14–56	20.1 ± 6.6	5–37	9.1 ± 3.6	2–18

**Table 6:** The mean plant height, mean length of the stem section with axillary bulbils and mean number of bulbils of mature flowering *Cardamine bulbifera* individuals in the studied populations. Significant differences between populations according to the Mann–Whitney test results (*p* < 0.05) are indicated by different letters in superscript across the same column.

Population	Number of Individuals (*n*)	Plant Height (cm)	Range (Min–Max; cm)	Length of Stem Section with Bulbils (cm)	Range (min–Max; cm)	Number of Bulbils	Range (Min–Max)
Kaukinė	5	52.8 ± 12.6 ^ac^	41–71	27.0 ± 10.4 ^a^	16–42	15.8 ± 1.9 ^ac^	13–18
Pašuliai	3	51.7 ± 4.7 ^ac^	48–57	29.3 ± 5.5 ^a^	24–35	18.3 ± 3.0 ^ab^	15–21
Šešuva	24	48.3 ± 8.1 ^a^	22–59	29.2 ± 4.8 ^ab^	21–39	13.6 ± 3.7 ^c^	4–21
Sutartiškės	6	50.3 ± 8.3 ^ac^	38–62	26.3 ± 8.2 ^a^	12–35	14.6 ± 3.0 ^ac^	9–18
Kalesninkai	39	54.3 ± 9.8 ^bc^	28–75	32.6 ± 7.2 ^c^	14–44	7.6 ± 3.8 ^d^	2–17
Vidzgiris	38	51.5 ± 8.9 ^ac^	24–67	29.2 ± 6.0 ^ad^	13–38	15.2 ± 3.2 ^ac^	6–21
Pooled data	115	51.8 ± 9.2	22–75	30.1 ± 6.7	12–44	12.4 ± 4.9	2–21

**Table 7:** The mean length of the inflorescence and the mean number of flowers of mature flowering *Cardamine bulbifera* individuals in the studied populations. Significant differences between populations according to the Mann–Whitney test results (*p* < 0.05) are indicated by different letters in superscript across the same column.

Population	Number of Individuals (*n*)	Length of Inflorescence (cm)	Range (Min–Max; cm)	Number of Flowers	Range (Min–Max; cm)
Kaukinė	5	2.3 ± 0.5 ^a^	2–3	3.2 ± 2.3 ^ab^	1–7
Pašuliai	3	2.0 ± 0.0 ^a^	2–2	3.3 ± 0.6 ^ab^	3–4
Šešuva	24	2.0 ± 0.6 ^a^	1–3	4.6 ± 1.5 ^a^	3–8
Sutartiškės	6	2.1 ± 0.7 ^a^	1–3	4.5 ± 1.4 ^a^	2–6
Kalesninkai	39	3.5 ± 1.4 ^b^	1–7	4.9 ± 2.6 ^a^	1–11
Vidzgiris	38	1.9 ± 1.0 ^a^	1–4	3.0 ± 1.5 ^b^	1–6
Pooled data	115	2.5 ± 1.2	1–7	4.1 ± 2.1	1–11

## Data Availability

All summarised data are presented in the article, and the authors can provide the original research data upon individual request and under agreed conditions.

## Appendix A

**Table A1:** The agrochemical characteristics of the soil at the study sites of *Cardamine bulbifera* populations.

Site	pH	P_2_O_5_ (mg/kg)	K_2_O (mg/kg)	N _total_ (mg/kg)	Humus (%)
Šešuva	6.2 ± 0.2	22 ± 4	68 ± 6	3.68 ± 0.46	7.26 ± 0.78
Pašuliai	4.8 ± 0.2	34 ± 5	39 ± 3	0.87 ± 0.26	2.63 ± 0.43
Sutartiškės	4.2 ± 0.2	8 ± 2	64 ± 5	0.87 ± 0.25	4.45 ± 0.57
Kaukinė	3.9 ± 0.2	35 ± 5	88 ± 7	1.94 ± 0.34	6.78 ± 0.74
Kalesninkai	5.5 ± 0.2	35 ± 5	241 ± 21	3.25 ± 0.43	4.52 ± 0.57
Vidzgiris	6.2 ± 0.2	22 ± 4	68 ± 6	3.68 ± 0.46	7.26 ± 0.78
